# Catheterization of Stenon's duct for surgical excision of oral fibroepithelial hyperplasia

**DOI:** 10.1590/S1808-86942012000100023

**Published:** 2015-10-20

**Authors:** Deygles Cristiane Barbosa, Rui Medeiros Júnior, Elaine Judite Amorim Carvalho, Alessandra Albuquerque Tavares de Carvalho

**Affiliations:** a Specialist. Dental surgeon; b Specialist in oral and maxillofacial surgery and traumatology. Oral and maxillofacial surgeon; c Doctoral degree in oral pathology. Assistant professor of oral pathology, UFPE.; d Doctoral degree in stomatology. Assistant professor of stomatology, UFPE. Pernambuco Federal University (Universidade Federal de Pernambuco or UFPE)

**Keywords:** hyperplasia, mucosa bucal, salivary ducts

## INTRODUCTION

Hyperplasias are the most frequent exophytic lesions in the mouth, which develop mainly as a consequence of mucosal tissue irritation by several types of trauma. Injured tissues respond with proliferation of fibroblasts followed by collagen fibrinogenesis^1^.

Fibroepithelial hyperplasia (FH) is the most common non-malignant soft tissue tumor in the mouth; its prevalence is similar in both sexes and there is no racial preference^2^. Its usual site is the jugal mucosa along the occlusion line; it presents as a well-defined smooth pink sessile or pediculated nodule. These tumors are usually asymptomatic except when injured secondarily^3^.

The treatment is surgical excision and elimination of local irritative factors. The purpose of this study was to describe a surgical technique for removing oral FH and preserving Stenon's duct.

## CASE REPORT

A male patient aged 36 years presented with an enlarged mass in the right jugal mucosa. The tumor had developed over about 13 years; it was painless at first, but the patient had started to feel pain when applying pressure over the nodule or during chewing.

**Figure 1:**
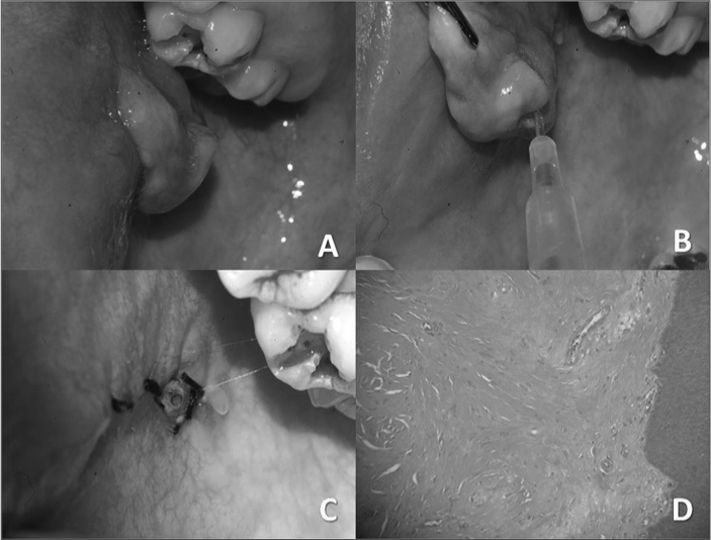
A) Clinical image of the tumor, showing its site next to the parotid duct exit point. B) Site of Stenon's duct - Jelco number 20. C) Final aspect after removal of the tumor; note the anterior portion of the Jelco in the exit point of the duct. D) Microscopy of the tumor showing fibroblasts dispersed within a collagen matrix and lined by squamous epithelium.

Examination of the mouth showed a hyperplasic pediculated fibrous pinkish smooth surfaced tumor measuring about 2.0 × 1.8 cm, located on the right jugal mucosa close to the exit site of Stenon's duct (Fig. 1A). Because of its close anatomical relation with the parotid duct, surgical removal would have to preserve this structure.

The treatment consisted of catheterizing the parotid duct with a Jelco number 20 catheter (Fig. 1B), followed by an elliptic incision of the tissue, dissection, removal of the tumor, and simple closure (Fig. 1C).

Histology revealed dense fibrous connective tissue with fusiform fibroblasts dispersed within a mature collagen matrix. There were many congested vascular spaces and mild chronic inflammation. The tumor was lined with hyperplasic epithelial squamous tissue (Fig. 1D).

The catheter was kept in place for seven days to activate salivary drainage; there were no postoperative complications or recurrences in the first year of follow-up.

## DISCUSSION

Lewkowicz at al.^4^ also reported catheterizing Stenon's duct when repairing trauma in the parotid region; these authors used a pediatric intravenous catheter, which makes it possible to locate where the duct courses and thereby avoid injury to the duct.

Parotid duct injury may result in sialoceles, cutaneous fistulae, or salivary duct cysts. The most common causes of duct injury are trauma by cutting, pointed, and blunt objects, as well as surgical trauma^5^.

Several treatment approaches have been described in the literature; the choice depends on the duration of injury, the site of the gland, the mechanism of trauma, and the surgeon's experience. Ideally parotid duct injury should be treated as soon as possible; in some cases (significant loss of tissues or polytraumatized patients) a delayed procedure may be done intentionally^6^.

Conservative approaches are repeated percutaneous needle aspiration, compressive dressings, and antisialagogue medication. A few authors also recommend parenteral nutrition to reduce autonomous salivary stimulation of the parotid^7^.

A more aggressive approach is needed if these measures are unsuccessful. These maneuvers include placing tubes in the site to create a new salivary duct path, radiotherapy, partial or total parotidectomy, tympanic neurectomy, and others^8^.

In the present case, after the duct was located and the tumor was removed, the anterior portion of the Jelco catheter was left in place during a week to facilitate salivary drainage and to avoid the sublingual caruncule closure and duct stenosis during the healing process.

The treatment for FH is surgery, taking care to remove the entire tumor and to avoid irritative factors to prevent recurrence^9^.

## FINAL COMMENTS

FH is one of the most common tumors in the mouth; it deserves attention when treated, as an adequate surgical technique is essential to avoid recurrences. Adjacent anatomical structures should be preserved as much as possible to avoid injury and future complications.
